# Supplementary figures and images for: Extended Evaluation of Viral Diversity in Lake Baikal through Metagenomics
Source: Microorganisms. 2021 Apr 5;9(4):760. doi: 10.3390/microorganisms9040760 (PMC8066274; doi:10.3390/microorganisms9040760)

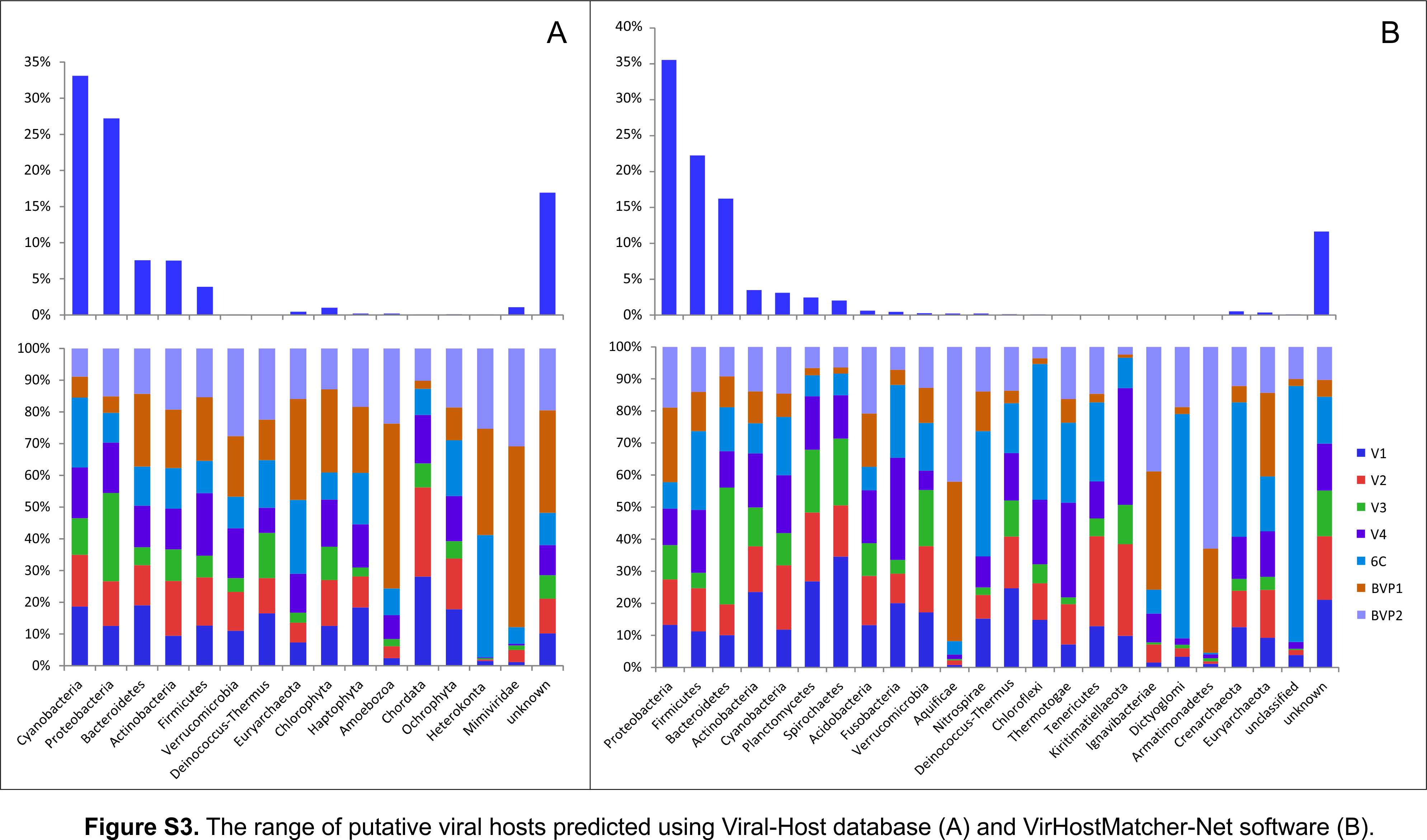

Supplement: Supplementary file 1 [file microorganisms-09-00760-s001.zip › Figure S4.jpg]

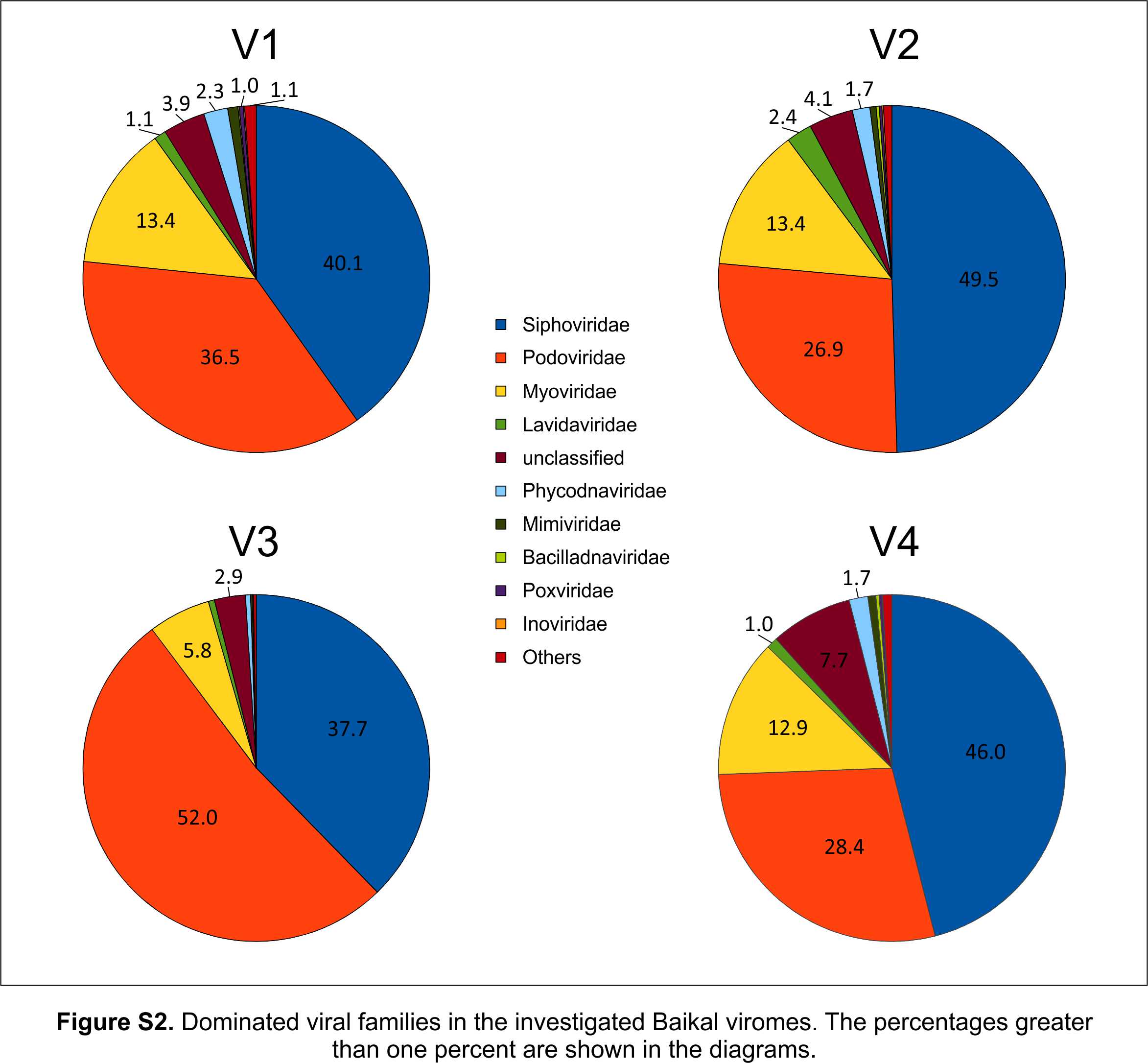

Supplement: Supplementary file 1 [file microorganisms-09-00760-s001.zip › Figure S2.jpg]

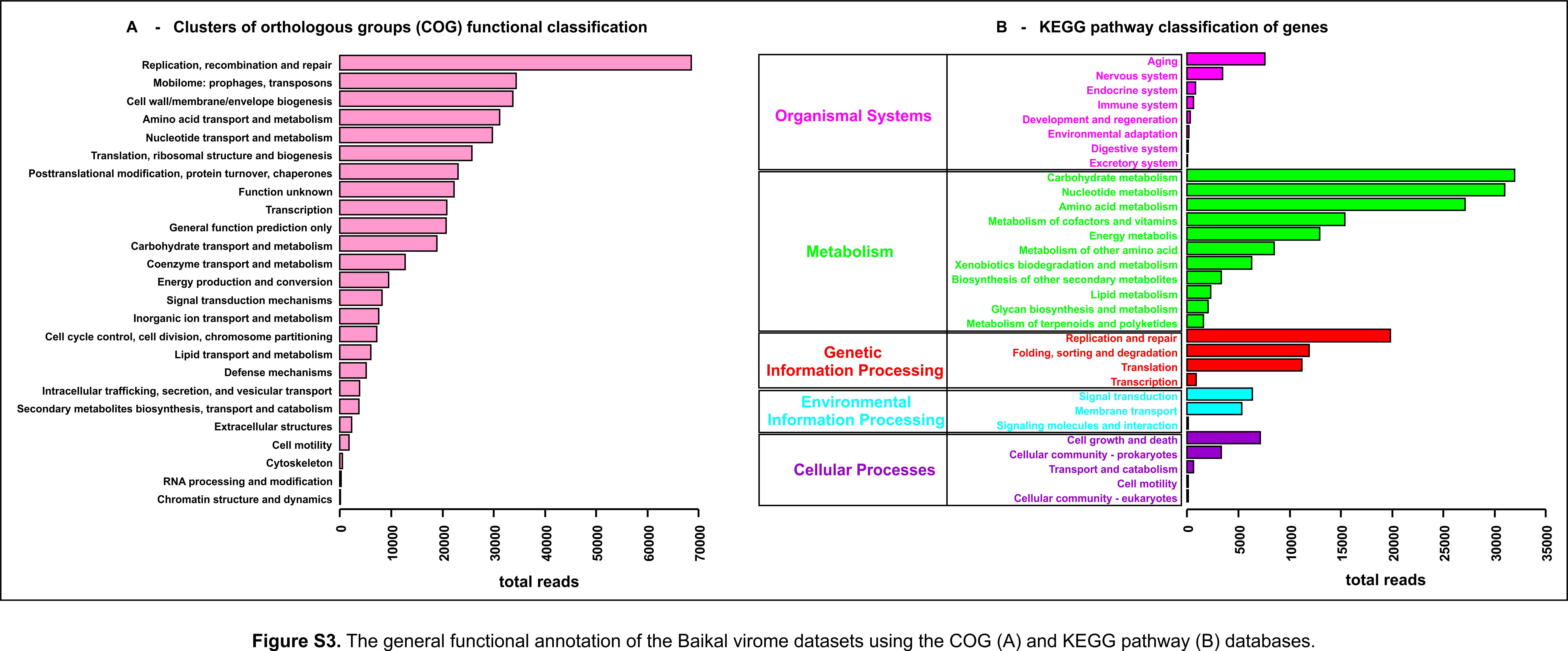

Supplement: Supplementary file 1 [file microorganisms-09-00760-s001.zip › Figure S3.jpg]
